# Draft genome sequence of *Halomonas* sp. SSL-5, a halophilic Mn(II)-oxidizing, perchlorate-tolerant bacterium

**DOI:** 10.1128/mra.01028-23

**Published:** 2024-01-05

**Authors:** Cameron J. Lee, Anthony C. Greene

**Affiliations:** 1 School of Environment and Science, Nathan Campus, Griffith University, Brisbane, Queensland, Australia; University of Southern California, Los Angeles, California, USA

**Keywords:** genome analysis, halophiles, manganese-oxidizing, perchlorate-tolerant

## Abstract

*Halomonas* sp. SSL-5 is a Mn(II)-oxidizing, perchlorate-tolerant halophilic bacterium isolated from an Australian hypersaline lake. The genome sequence contains 27 contigs, and the genome is 3.4 Mb with a GC content of 67.2%. The sequence provides information for future studies of Mn(II) oxidation and perchlorate resistance under halophilic conditions.

## ANNOUNCEMENT

Microbial manganese(II) oxidation is an aerobic process transforming Mn(II) to Mn(IV) oxides, which occurs in aquatic and terrestrial environments at much higher magnitudes compared to chemical oxidation (1, 2). While the mechanisms are well studied among marine and freshwater systems at near neutral pH, to date, there have been only a couple of studies investigating halophilic Mn(II) oxidation (3, 4). In addition, some halophiles are capable of tolerating perchlorate levels up to 0.4 M (5). The aim of this work was to understand the molecular mechanisms associated with Mn(II) oxidation and perchlorate tolerance under moderate halophilic conditions.

*Halomonas* sp. strain SSL-5 was isolated from a 500-g hypersaline water sediment sample collected at a 50-cm depth from the shoreline of Streatham Salt Lake (37°46′10.269″S, 143°6′15.333″E) in Victoria, Australia, in October 2019 (stored at 4°C until used). Enrichment cultures were established by adding 5 g of sediment to 200 mL of tryptone (5 g/L), yeast extract (3 g/L), and glucose (1 g/L) (TYEG) broth supplemented with 1.7 M NaCl, 1 mM MnCl_2_·4H_2_O, and 40 mM NaClO_4_. The strain was isolated from a positive Mn(II)-oxidizing enrichment using streak plating in the same medium solidified with 16 g/L bacteriological agar. A single colony that was positive for Mn(II) oxidation (dark brown) was picked and subcultured to obtain a pure culture. Mn(II) oxidation was verified using the leucoberbelin blue test (6).

For genome sequencing, strain SSL-5 was grown in TYEG broth (Oxoid) supplemented with 1.7 M NaCl for 24 h on an orbital shaker at 27°C. Purified DNA was extracted using a Wizard Genomic DNA Purification Kit (Promega), according to the manufacturer’s instructions. Short-read libraries were prepared using the Nextera DNA Flex Library Preparation Kit (Illumina) and pooled at equimolar amounts of 2 nM/library. Sequencing was conducted with the Novaseq 6000 platform using SP Reagent Kit v.1.5 (Illumina) and 2 × 150 bp paired-end chemistry. This resulted in 10,438,880 reads, equating to 1.36 Gbp of data. The quality of the sequencing data was assessed using FastQC v.0.11.7 (7). Default parameters were used for all software unless otherwise specified. Trimming and correction of the sequences were performed using Trimmomatic v.0.36 (8) with PhiX sequences removed by BBMap v.38.32 (9). Quality sequences were assembled with SPAdes v.3.13.0 specific for Illumina reads (10). Annotation was performed using Prokaryotic Genome Annotation Pipeline v.6.5 (11). Protein-coding sequences were predicted by Prodigal 2.6.2 (12). Taxonomic identification was conducted using the average nucleotide identification (ANI) platform Orthologous ANI Tool (OAT) v.0.93.1, a digital DNA–DNA hybridization tool (13).

*De novo* assembly of strain SSL-5 produced 27 contigs with 359× coverage. The largest contig was 406,988 bp, and the N50 was 266,268 bp. The assembly possessed a GC content of 67.2% and was 3,460,514 bp in length. Annotation suggested that the assembly contained 3,180 genes, 3 complete rRNA genes (1 × 5S, 1 × 16S, and 1 × 23S), and 58 tRNAs. OAT suggests that strain SSL-5 aligns closest to *Halomonas alimentaria* (DSM 15356, GenBank assembly accession number GCA_009902005.1) with an ANI value of 92.24%.

## Data Availability

The genome sequence and annotation data for *Halomonas* sp. SSL-5 were deposited in GenBank under BioProject number PRJNA956999, BioSample number SAMN34238072, SRA number SRX20017196, SRR number SRR24220786, and accession number JAUYWF000000000. The version described in this paper is the first version, JAUYWF010000000.
